# Development of the Navigation Guide Evidence-to-Decision Framework for Environmental Health: Version 1.0

**DOI:** 10.1021/acs.est.4c08063

**Published:** 2025-02-27

**Authors:** Nicholas Chartres, Max T. Aung, Susan L. Norris, Courtney Cooper, Lisa A. Bero, Roger Chou, Devon C. Payne-Sturges, Wendy E. Wagner, Jessica W. Reyes, Lisa M. Askie, Daniel A. Axelrad, Deysi Flores Vigo, Jill E. Johnston, Juleen Lam, Keeve E. Nachman, Eva Rehfuess, Rachel Rothschild, Patrice Sutton, Lauren Zeise, Tracey J. Woodruff

**Affiliations:** † Program on Reproductive Health and the Environment, Department of Obstetrics, Gynecology, and Reproductive Sciences, University of California, San Francisco, California 94143, United States; ‡ School of Pharmacy, Faculty of Medicine & Health, The University of Sydney, Sydney 2006, Australia; § Department of Population and Public Health Sciences, Keck School of Medicine, University of Southern California, Los Angeles, California 90033, United States; ⊥ Department of Family Medicine, School of Medicine, Oregon Health & Science University, Portland, Oregon 97239, United States; ¶ Center for Bioethics and Humanities, University of Colorado Anschutz Medical Campus, School of Medicine and Colorado School of Public Health, Aurora, Colorado 80045, United States; □ Department of Medicine, School of Medicine, Oregon Health & Science University, Portland, Oregon 97239, United States; ■ Department of Global, Environmental, and Occupational Health, University of Maryland School of Public Health, College Park, Maryland 20742, United States; ○ The University of Texas at Austin School of Law, Austin, Texas 78705, United States; ● Department of Economics, Amherst College, Amherst, Massachusetts 01002, United States; △ NHMRC Clinical Trials Centre, The University of Sydney, Sydney 2006, Australia; ▽ Make the Road New York, Brooklyn, New York 11237, United States; ▼ Department of Public Health, California State University, East Bay, California 94542, United States; ⬡ Department of Environmental Health and Engineering, Johns Hopkins Bloomberg School of Public Health, Johns Hopkins Risk Sciences and Public Policy Institute, Baltimore, Maryland 21205, United States; ⬢ Institute for Medical information Processing, Biometry and Epidemiology, Faculty of Medicine, LMU; Pettenkofer School of Public Health, Munich 3608, Germany; ⇌ University of Michigan Law School, Ann Arbor, Michigan 48109, United States

**Keywords:** Evidence to Decision Framework, Rule Making, Recommendations, Risk Management, Health Equity, Environmental Justice, Essentiality, Risk Assessment

## Abstract

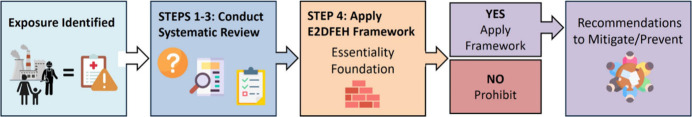

Environmental exposures, including widespread industrial pollution, impact human health and are amplified in more highly exposed communities. Policy and regulatory frameworks for making decisions and recommendations on interventions to mitigate or prevent exposures tend to narrowly focus on exposure and some health-related data related to risks. Typically, such frameworks do not consider other factors, including essentiality, health equity, and distribution of benefits and costs. Further, decisions and recommendations lack transparency regarding how they were developed. We developed the Navigation Guide Evidence-to-Decision Framework for Environmental Health (E2DFEH) to provide a structured and transparent framework incorporating a range of scientific information and factors for decision-making. We reviewed current evidence-to-decision frameworks and engaged in an iterative consensus-based process involving 30 experts from 25 organizations in the academic, government, and nonprofit sectors. The E2DFEH framework includes three Foundations that are structural factors considered as part of recommendation development: 1) Essentiality, 2) Human Rights, and 3) Quality of the Evidence. It also includes three core Criteria that guide the development of a specific recommendation, informed by an evaluation of relevant evidence: 1) Environmental Justice, 2) Maximizing Benefits and Reducing Harm, and 3) Sociocultural Acceptability and Feasibility. The framework’s goal is to make the decision process transparent and comprehensive through explicit consideration of core factors important for decisions, leading to more equitable and health-protective interventions.

## Abstract 2

This framework provides a transparent and consistent approach for developing recommendations to protect human health from environmental exposures and address inequitable community impacts

## Introduction

Ongoing and emerging environmental exposures such as chemical pollution, climate change, and natural resource extraction pose major health risks to populations.^1−4^ Chemical pollution has now crossed a “planetary boundary” with over 350,000 chemicals registered for production and use globally, with only a fraction assessed for safety.^5,6^ The World Health Organization (WHO) estimates that two million lives were lost due to chemical exposures in 2019. This estimate is based on methods that do not fully capture all possible risks and is thus likely an underestimate.^7^

Across the world and in the United States (U.S.), these health effects are amplified in communities that are marginalized due to multiple interacting factors such as systemic racism, historical residential segregation, geographic placement of polluting facilities, targeted marketing of toxic products and inequitable access to quality education, healthcare or healthy food.^8−10^ For example, approximately 134 million Americans living within “vulnerability zones,” which surround industrial facilities that produce, store, or use highly hazardous chemicals, are predominantly Latinx or African American, with relatively high rates of poverty and low levels of income and educational attainment.^11^

Current approaches to identifying and preventing widespread population exposure to harmful chemicals have lagged behind chemical production and use. This is in part due to structural approaches to the regulation of chemicals in the U.S., including a legal and regulatory system in which safety is assumed until harm is proven, lack of legal requirements for full disclosure of where chemicals are present and their potential adverse health effects, industry influence, and suboptimal methods used to capture risks from chemical exposures which do not incorporate current scientific knowledge for hazard and risk assessment.^12−16^ Apart from selected agents with extensive epidemiological evidence such as particulate matter and lead, historically, there has been little formal consideration of factors critical for equitable decision-making including full accounting of health impacts at different levels of exposure, distributions of harm, and considerations of community concerns. For example, the U.S. Environmental Protection Agency (EPA) does not generally quantify the expected health effects from chemical exposure (e.g., proportion of an exposed population anticipated to experience effects at different exposure levels) of harms that are not cancer, such as reproductive, metabolic, and neurodegenerative, or for exposure/health outcomes considered more uncertain (e.g., hazard classification of “suggestive”). Thus, the current approaches for considering and quantifying benefits for EPA proposed regulations in most cases do not include health effects other than cancer, which underestimates the benefits.^16^ Further, the EPA traditionally has focused on aggregate health benefits from prevention or mitigation of a hazardous exposure without considering the distribution of those benefits by factors such as race/ethnicity or socioeconomic status; though EPA’s Guidelines for Preparing Economic Analyses notes that it is important to consider distributional analyses and provides examples of how this can be conducted.^17−19^ There are recent examples of EPA analyzing the distribution of exposure, health risk and risk reductions in analyses supporting regulations of air emissions from chemical manufacturing and perchloroethylene under the Toxic Substances Control Act (TSCA).^20,21^ In 2023, the Biden administration issued updated guidance for regulatory analysis that outlined issues to consider in assessing the distribution of regulatory benefits and costs. However, conducting this type of analysis remains optional under the updated guidance.^19^

Thus, there is a need for a structured and transparent framework that facilitates timely decision-making based on a range of scientific information and other important considerations. Evidence-to-decision (EtD) frameworks for interventions in clinical medicine and public and environmental health provide a structure for decision-making and include explicit considerations relevant to a decision context.^22^ That context might be improving patient care, optimizing health systems, or protecting individuals or communities from historic or emerging hazardous exposures. EtD frameworks can be used by decision-makers, including regulators and guideline panels. Presentation of quantitative or qualitative evidence for each consideration guided by signaling questions facilitates a transparent and comprehensive decision-making process. This includes articulating the pros and cons of potential interventions, identifying reasons for any disagreements among decision-makers, and crafting the rationale statement for each recommendation. This process can also facilitate structured input from diverse perspectives, including intended end users such as regulators, community and clinical members who can inform considerations such as feasibility and acceptability of the intervention.^22^ End-users can review the considerations in the framework and understand the many considerations and perspectives that informed the recommendations when making decisions on adoption or adaptation of recommendations to specific settings.^22^

We have previously reviewed EtD frameworks for decision-making in environmental health and identified multiple frameworks.^22^ Fourteen organizations including GRADE (Grading of Recommendations, Assessment, Development and Evaluation) Working Group, five agencies of national governments, one U.S. state agency (California Environmental Protection Agency), two related to the WHO and the remainder are nongovernmental organizations or academic groups) provided 18 EtD frameworks; most focused on clinical medicine or public health interventions; four on environmental health and three on economic considerations. We identified several limitations that make it difficult to apply these frameworks to environmental health. A key limitation is that they do not articulate considerations or criteria for making decisions, making current frameworks difficult to apply and leading to divergent decisions and conflicting recommendations. We also identified key criteria that either were not included in the frameworks or were not sufficiently emphasized or described that are critical to decision-making in environmental health. These include the essentiality of a hazardous (chemical) agent (whether the chemical of concern is considered essential for health and safety, or for societies to function),^23,24^ the explicit and prioritized consideration of environmental justice (i.e., consideration of unequitable distribution of risks/health outcomes both contemporary and historically) and the distribution of the benefits of an intervention (i.e., ensuring equitable distribution of the benefits/harms of interventions).^22^ The two most comprehensive and well-developed existing EtD frameworks are GRADE^25^ and WHO-INTEGRATE.^26^ While these frameworks can be used for decision-making with multicomponent interventions and in complex contexts, they do not sufficiently emphasize or clearly articulate considerations that are highly relevant to decision-making in environmental health policy, such as essentiality and environmental justice.^22^

This paper presents our work developing the Navigation Guide Evidence-to-Decision Framework for Environmental Health (E2DFEH), which is a tool to help decision-makers, including regulators and guideline panels (which include representatives from impacted communities), use a transparent and consistent approach to considering factors that are important for developing intervention recommendations to protect human health. This framework recognizes the vision of the Louisville Charter, and the fundamental, comprehensive reform necessary to protect high-risk and highly exposed communities and the environment from the cumulative effects of industrial chemicals.^27^ The Louisville Charter is a roadmap and set of recommendations to advance environmental justice in communities disproportionately impacted by harmful and cumulative chemical exposure and, when adopted, will achieve a safe and sustainable chemical industry that does not harm people, the environment, or the climate. It was devised by a coalition of grassroots, labor, health, and environmental justice groups. The E2DFEH framework is intended to be broadly applicable to environmental hazards globally and at different levels of government from local to state and national, including regulators, the community, and clinical members.

## Materials and Methods

### Development of the Evidence-to-Decision Framework for Environmental Health

The Program on Reproductive Health and the Environment (PRHE), University of California, San Francisco (UCSF), developed the Navigation Guide, which is a methodology for conducting transparent and rigorous systematic reviews of human and animal evidence on the health effects of hazardous chemicals.^28−34^ The Navigation Guide methodology leads to the development of a concise summary of the strength of evidence on health effects of chemicals that can be used to develop recommendations on interventions that prevent or mitigate harmful chemical exposures.^30^ To date, however, the Navigation Guide has not encompassed guidance on how to develop such recommendations.

**Figure 1:**
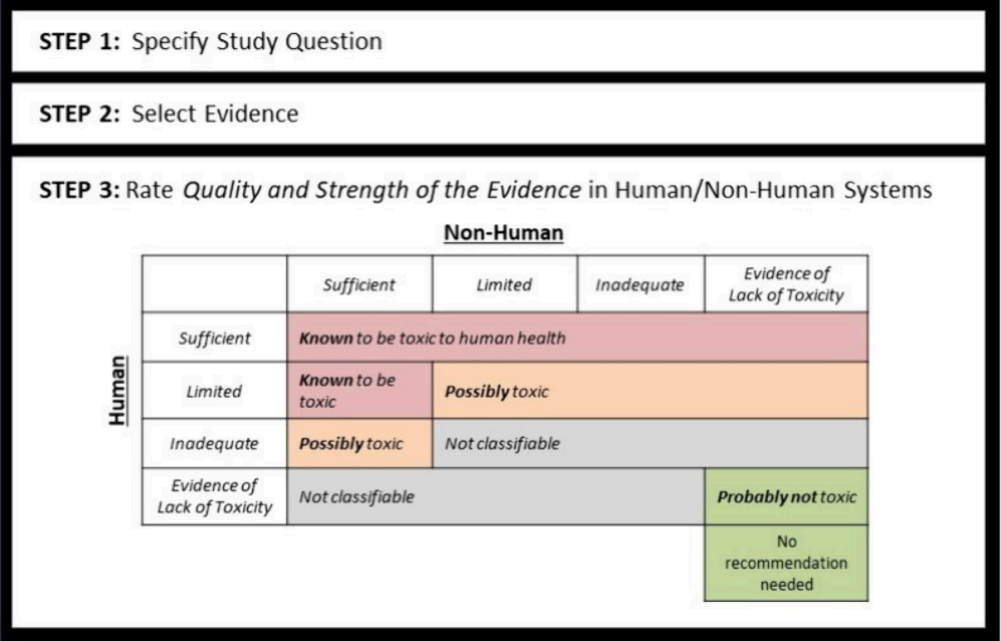
Navigation Guide Systematic Review Method. The Navigation Guide method, including a description of how the quality and strength of evidence is assessed is available in previous publications, including systematic reviews.^28^

The Navigation Guide E2DFEH used the Navigation Guide systematic review methods^30^ as the starting point to guide conceptualization and development (Figure 1). Once the strength of the evidence on the human health effects of a hazardous chemical has been rated and there is sufficient or adequate evidence to demonstrate harm, for example, “known to be toxic” or “possibly toxic”” (Figure 1. Step 3), the Navigation Guide proposes a fourth step of formulating recommendations. Recommendations are focused on interventions to prevent or mitigate the exposure based on a range of considerations.

As a first step in developing the E2DFEH, we conducted a scoping review of existing EtD frameworks used in clinical medicine, public health, and environmental health.^22^ This review identified 18 EtD frameworks from 14 organizations, which yielded a list of potential criteria that could be considered in decision-making and identified gaps in criteria most relevant to environmental health (the methods and results are provided in Norris et al. 2021).^22^ While the GRADE EtD framework and WHO-INTEGRATE provided a useful starting point, we determined that additional criteria were needed. As well, we noted that criteria in existing frameworks did not sufficiently emphasize factors that are critical for equitable decision-making in environmental health. We, therefore, concluded that a new framework for environmental health decision-making was necessary–one that built on existing work. This has become particularly salient, as the EPA has recently been issuing new proposed regulations based on the 2016 amendments to TSCA that can account for factors in addition to the science.

The next step in our development process was to identify and assemble a multidisciplinary Steering Committee consisting of six members encompassing a broad range of expertise with diverse perspectives and interests. This included expertise in environmental health, environmental justice, evidence synthesis methods, clinical medicine, law, economics, and guideline development. The Steering Committee members had extensive experiences in working in academia, nongovernmental international health agencies, including the WHO, national governmental health agencies, including EPA, government departments, including Baltimore City Health Department, and medical facilities and institutions, including the Internal Medicine Clinic at Oregon Health and Science University.

The Steering Committee met three times (May, August, and November 2021) to develop the draft E2DFEH and provide recommendations about the structure and components via an iterative, consensus-based process. In the first meeting, the findings of the scoping review were presented, followed by a discussion of the strengths and limitations of the frameworks. Emerging themes from the meeting included the need to consider health impact assessments and the challenges of collecting data to inform environmental health decision-making. In the second meeting, the WHO-INTEGRATE framework was presented, and the Steering Committee discussed the value of criteria specifically for environmental health decision making and how the subcriteria could be modified. Emerging themes from the meeting included the need to consider human rights and equity, as does WHO-Integrate and to prioritize environmental justice. In the third meeting, a draft set of framework criteria, subcriteria, signaling questions, and considerations was presented, that reflected the emerging themes from the second meeting. Following in-depth discussion, the Steering Committee then provided feedback, and a draft framework was agreed upon.

The next step was to collect feedback on the draft E2DFEH from diverse scientists, legal experts, and community-based representatives. We hosted a two-day workshop in March 2022 with 30 experts from 25 different organizations in the academic, government, and nonprofit sectors. Nineteen experts were from academia, three from U.S. government (federal and state) health agencies, three from nonprofit advocacy groups, two from nongovernmental organizations, two independent consultants, and one from a nongovernmental health agency. During the workshop, we introduced the rationale for developing the framework and presented a summary of the prior Steering Committee meetings. We collected anonymous feedback from each attendee via reporting boards and incorporated these comments into the framework. We then drafted a manuscript with the updated framework and circulated it for further input from both the Steering Committee and the workshop participants.

## Results

### The Evidence-to-Decision Framework for Environmental Health

**Figure 2:**
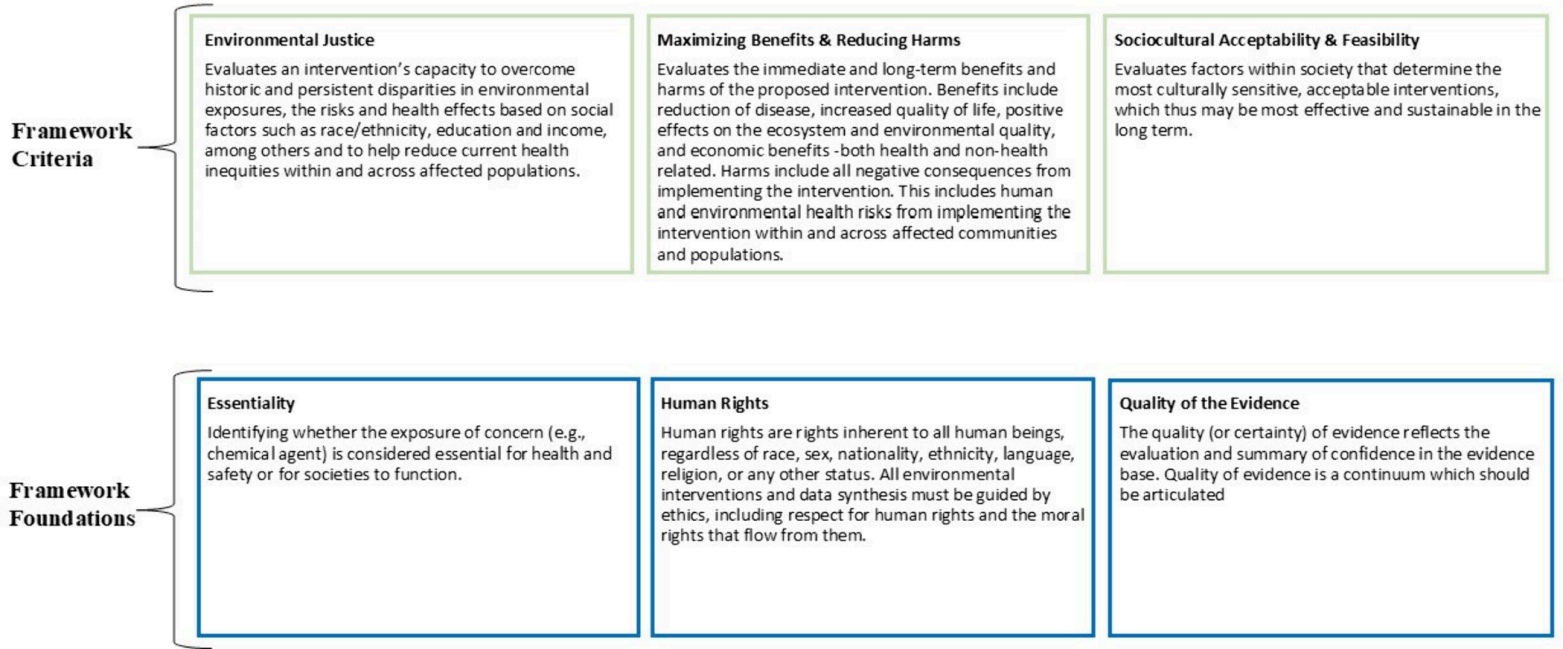
Navigation Guide Evidence-to-Decision Framework for Environmental Health Foundations and Criteria.

The E2DFEH finalized by the Steering Committee and workshop participants contains two basic parts: “Foundations” and “Criteria” (Figure 2). *Foundations* are structural factors that must be considered as part of the development of all intervention recommendations either at the beginning of the decision-making process or when considering each individual Criterion.

**Figure 3:**
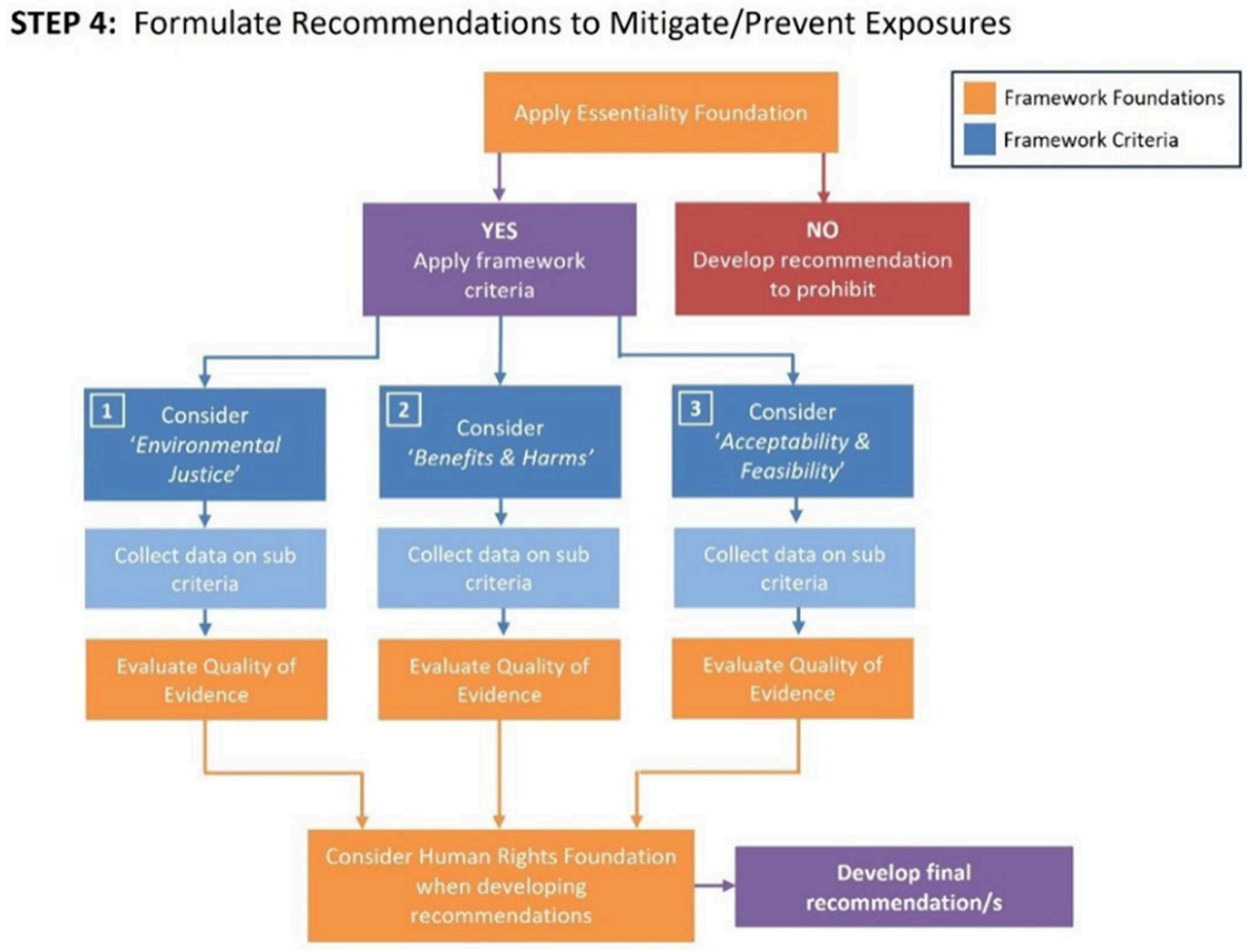
Navigation Guide Evidence-to-Decision Framework for Environmental Health (E2DFEH), Step 4.

They include Essentiality (considered at the beginning of the process), Human Rights, and Quality of the Evidence (both considered when using the criteria to develop the recommendations). *Criteria* are core factors that guide the development of a specific recommendation, which are informed by an evaluation of relevant evidence. They include Environmental Justice, Maximizing Benefits and Reducing Harms, and Sociocultural Acceptability and Feasibility.

(See Figure 3 to see how the *Foundations* and *Criteria* are considered in the decision-making process for developing intervention recommendations)

**Figure 4:**
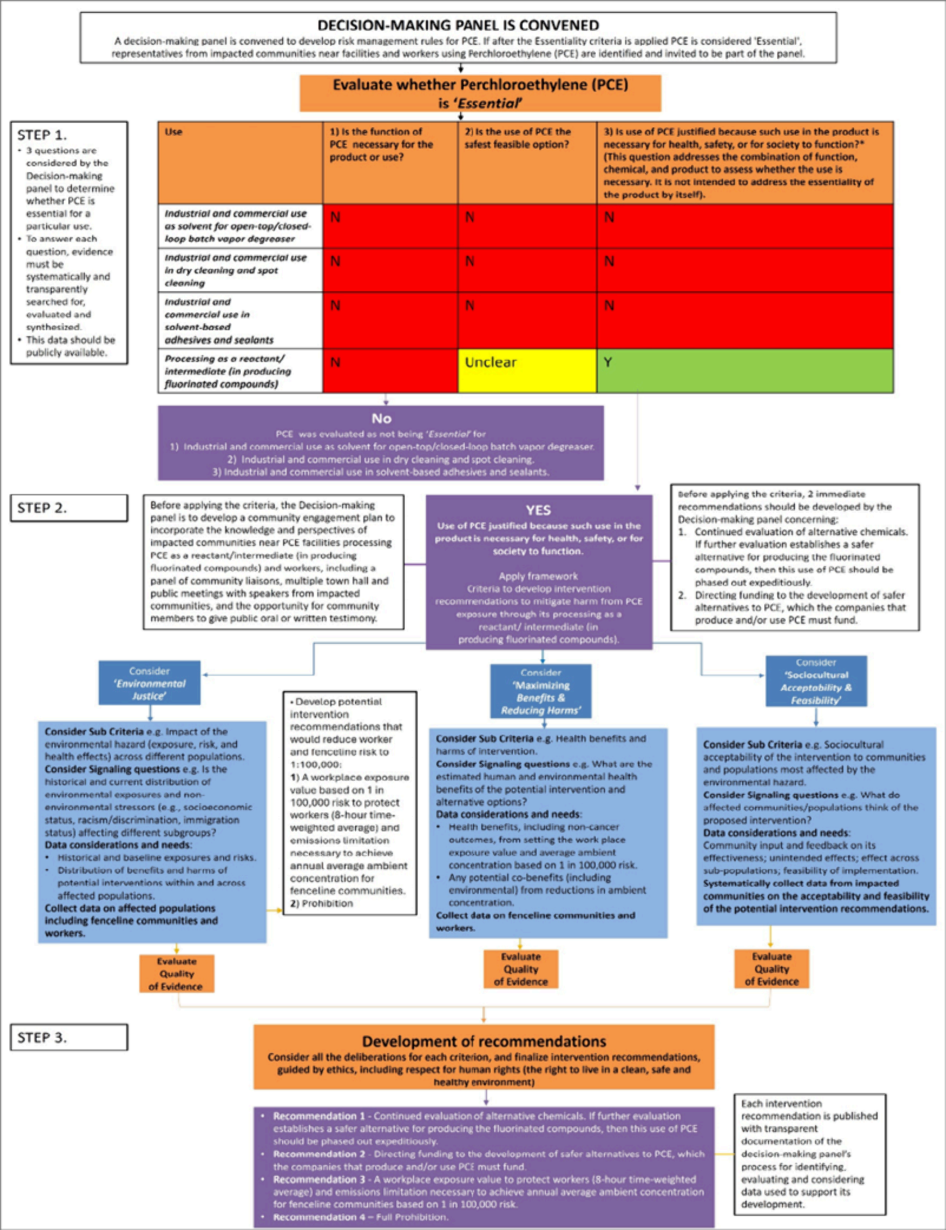
Hypothetical example of how the Navigation Guide Evidence-to-Decision Framework for Environmental Health would be operationalized when developing intervention recommendations for perchloroethylene (PCE).

We present in Figure 4. a hypothetical example of how the E2DFEH would be operationalized when developing intervention recommendations for the hazardous solvent perchloroethylene (PCE) by a decision-making panel. EPA has recently completed a risk evaluation under TSCA for PCE and is finalizing risk management rules. EPA determined PCE presents an “unreasonable risk” to human health.^35^ EPA’s final risk evaluation found that 60 of the 61 conditions of use (COUs) EPA evaluated create an “unreasonable risk” as part of their determination.^35^ The unreasonable risk finding triggered a mandatory risk management process, and EPA issued a proposed regulation in June 2023.^36^ The risk management actions or interventions that EPA proposed varied by PCE COU and included: a ban, labeling requirements, applying an existing chemical exposure limit to occupational exposures, among others.

### Framework Foundations

Three Foundations must be considered as part of the development of all intervention recommendations using the E2DFEH.

(See Table S1 in the Supporting Information for the “Navigation Guide Evidence-to-Decision Framework for Environmental Health Foundations and Considerations”).

#### Essentiality

Essentiality refers to identifying whether the exposure of concern (e.g., use of a chemical agent) is considered essential for health and safety, or for societies to function.^23,24^ Assessing essentiality is primary: if a chemical agent is essential, absolutely necessary, then the next step is to consider what harms are reasonable to incur in its use. If, on the other hand, a chemical agent is not essential, society need not accept a harm. Consequently, the essentiality is determined prior to operationalizing the framework.

##### Considerations

There are three considerations to determine whether an exposure of concern is essential for a particular use as outlined by Balan et al., 2023^23^ (Table S1, Supporting Information):

1) Is the function of the chemical necessary for the product or use?
2) Is the use of the chemical the safest feasible option? And,
3) Is use of the chemical justified because such use in the product is necessary for health, safety, or for society to function.

In the example in Figure 4. each of the COUs of PCE would be evaluated for each of the “Essentiality” criteria in step 1. While three of the uses are not determined to be essential (therefore the production of PCE for these uses should be stopped expeditiously), one COU (using PCE as a reactant/intermediate in producing fluorinated compounds) has an “unclear” determination about whether there are safer alternatives available. In this case, the decision-makers should 1) recommend alternative chemicals continue to be evaluated, and 2) funding is directed to the development of safer alternatives to PCE, which the companies that produce or use PCE must fund (Step 2). A five-year phase-out of the substance should be implemented, unless the industry establishes that after a reasonable investment in innovation, they were unable to identify a safer substitute. The Framework *Criteria* should then be applied to develop intervention recommendations to mitigate the harm from PCE exposure for this COU. If, however, safer alternatives are identified, the appropriate risk management decision is that production and use of PCE should be stopped expeditiously, and application of the Framework *Criteria* is not necessary to decide on a course of action.

#### Human Rights

Human rights are rights inherent to all human beings, regardless of race, sex, nationality, ethnicity, language, religion, or any other status.^37^ The WHO Constitution (1946) envisages “. . .the highest attainable standard of health as a fundamental right of every human being without distinction of race, religion, political belief, economic or social condition.”^38^ Environmental interventions and recommendations must be guided by ethics, including respect for human rights and the moral rights that flow from them. For environmental health interventions, the most important human right is the right to live in a clean, safe and healthy environment; in this includes rights of equal and equitable access to clean air, water, sufficient health-promoting food, and health.

##### Considerations

The framework criteria and subcriteria are firmly grounded in human rights, which encompass both the moral rights to not be exposed to harms, including chemical and nonchemical exposures, and affirmative rights to health (i.e., rights to survive *and* thrive). When developing recommendations, the decision–making panel must consider that the victims should have an adequate opportunity for “informed consent” of the risks and accompanying unknowns by the risk-creator.

(Table S1, Supporting Information and Figure 4. Step 3).

#### Quality of the Evidence for the Intervention or Prevention

The quality (or certainty) of evidence reflects the extent to which confidence in the body of evidence is adequate to support a particular recommendation about an intervention.^39,40^ Approaches to evaluating the quality of the body of evidence and comparing the efficacy and effectiveness of interventions are well established in the clinical sciences using the GRADE approach (and include consideration of various factors such as the risk of bias of the individual studies being considered, and imprecision and inconsistency across studies)^39^ and GRADE CERQual (Confidence in the Evidence from Reviews of Qualitative Research)^41^ is appropriate for evaluating qualitative data. The inferences that can be made about a body of evidence are stronger for higher quality evidence than for lower quality evidence. However, decisions can be made on any body of evidence, regardless of quality.

##### Considerations

Optimal approaches to gathering and evaluating the evidence differ for each of the three framework Criteria described below (section on Framework Criteria). Some criteria and subcriteria will be best informed by systematic reviews of quantitative human research evidence, while others may be best informed by qualitative research, including interviews or focus group discussions with diverse end-users, including community representatives. The results of economic analyses, such as the costs of interventions, may also be important to decision-making in some contexts. Additionally, there may be little or no direct evidence regarding interventions for a specific environmental exposure of concern; however, indirect evidence on the effectiveness of interventions for other exposures of concern may be informative. The quality of the body of evidence relevant to each framework criterion should be evaluated with validated tools and approaches when available (Table S1, Supporting Information and Figure 4. Step 2).

An essential component of evidence evaluation is an assessment of the risk of bias. Data produced by those with a financial stake in the outcome, including the polluting industry that manufacture, distribute or sell chemicals, should be carefully evaluated for risk for bias.^42^ This includes studies on health harm and analyses that include financial costs for implementation. Empirical evidence demonstrates that analyses conducted by those with a financial stake are biased in favor of the sponsor.^43−45^ Alternatively, often those harmed are subject to multiple overlapping disadvantages, which includes lack of access to research funding and study participation. Thus, those that are experiencing harm are frequently underrepresented in research, potentially biasing results.

### Framework Criteria

Three Criteria are used to guide the development of intervention recommendations using the E2DFEH: 1) Environmental Justice; 2) Maximizing Benefits and Reducing Harms; and 3) Sociocultural Acceptability and Feasibility (SeeTable S2, Supporting Information for the “Navigation Guide Evidence-to-Decision Framework for Environmental Health Criteria, Sub-Criteria, Signaling Questions, and Considerations”).

We developed signaling questions to guide the evidence-gathering process, which are based on those used in the WHO-INTEGRATE framework.^26^ Evidence for each criterion and subcriteria should be identified, evaluated, synthesized, and considered when crafting potential recommendations.

#### Environmental Justice

##### Definition and Description

*Environmental Justice* evaluates an intervention’s capacity to overcome historic and persistent disparities in environmental exposures; the risks and health effects based on social factors such as race/ethnicity, education, and income, among others; and to help reduce current health inequities within and across affected populations.

The EPA defines environmental justice as “the fair treatment and meaningful involvement of all people regardless of race, color, national origin, or income, with respect to the development, implementation, and enforcement of environmental laws, regulations, and policies. This goal will be achieved when everyone enjoys the same degree of protection from environmental and health hazards, and equal access to the decision-making process to have a healthy environment in which to live, learn, and work.”^46^

The U.S government has recognized and recommended consideration of environmental justice in decision making as per multiple Executive Orders, Memorandums and Initiatives.^a^ The *Executive Order Revitalizing Our Nation’s Commitment to Environmental Justice for All* directs agencies to “identify, analyze, and address historical inequities, systemic barriers, or actions related to any Federal regulation, policy, or practice that impair the ability of communities with environmental justice concerns to achieve or maintain a healthy and sustainable environment. . .provide opportunities for the meaningful engagement of persons and communities with environmental justice concerns who are potentially affected by Federal activities.”^47,48^

An important aspect of achieving environmental justice is addressing environmental racism. Environmental racism refers to “those institutional rules, regulations, and policies of government or corporate decisions that deliberately target certain communities for least desirable land uses, resulting in the disproportionate exposures of toxic (chemicals) and hazardous waste on communities based upon prescribed biological characteristics. Environmental racism is the unequal protection against toxic and hazardous waste exposures (inclusive of chemicals) and the systemic exclusion of people of color from decisions affecting their communities.”^49^

##### Signaling Questions and Data Considerations

We developed five signaling questions focused on reducing health inequities by prioritizing marginalized and affected communities (see Table S2, Supporting Information for the Navigation Guide Evidence-to-Decision Framework for Environmental Health Criteria, Sub-Criteria, Signaling Questions and Considerations):

- What is the historical and current distribution of environmental exposures and nonenvironmental stressors (e.g., socioeconomic status, racism/discrimination, immigration status) affecting different groups?
- What are the cumulative effects of all environmental exposures and nonenvironmental stressors?
- What is the expected/estimated reduction in exposure, risk, and health effects from the hazardous exposure following implementation of the intervention in groups that have been historically marginalized?
- How does the proposed policy address cumulative exposures and effects?
- Will the expected distributional consequences of the intervention (including as part of any analysis of benefits and harms) appropriately benefit and not inappropriately burden disadvantaged, vulnerable, or marginalized communities?

The signaling questions integrate principles that have been developed to address environmental racism, which include:

- That public policy should be based on mutual respect and justice for all peoples, free from any form of discrimination or bias;
- Universal protection from extraction, production, and disposal of toxics, hazardous wastes, and poisons that threaten access to clean air, land, water, and food; and
- The right to participate as equal partners at every level of public environmental decision making, including needs assessment, planning, implementation, enforcement, and evaluation.^50−53^

This criterion should also ensure that there are diverse and affected groups included in data collection, question assessment, and evaluation. Consultation or consideration of relevant data is essential when applying this criterion.

For example, if a decision-making panel was convened to develop risk management rules for PCE, it should include representatives from impacted communities near facilities producing or using PCE and workers should be identified and invited to be part of the panel (Figure 4, ‘Decision Making Panel is convened’). After the “Essentiality” criterion has been applied, and a decision has been made to apply the Framework to develop intervention recommendations, the Decision-making panel would develop a community engagement plan to incorporate the knowledge and perspectives of impacted communities and workers from the harms of PCE. This will provide impacted communities the opportunity to give public oral or written testimony on their lived experiences from being exposed to PCE (Figure 4, Step 2). The Decision-making panel would then apply the Environmental Justice criterion, considering the levels of exposure and risk of PCE across different groups of populations, including fenceline communities and workers (sub criteria), whether there are additional persistent and historical environmental (e.g., other chemicals) and nonenvironmental stressors (e.g., racism, poverty) affecting these impacted communities (signaling questions), and what data they require from the facility to fully quantify these baseline exposure and risks and to determine the potential benefits and harms of a proposed intervention to protect these impacted communities (Figure 4, Step 2, “Environmental Justice”). Potential intervention recommendations are then developed to satisfy TSCA’s requirement that all unreasonable risks are eliminated (e.g., 1-in 10,000 or 1-in-100,000 risk).

#### Maximizing Benefits and Reducing Harms

##### Definition and Description

*Maximizing Benefits and Reducing Harms* evaluates the immediate and long-term benefits and harms of the proposed intervention. Benefits include reduction of disease, increased quality of life, positive effects on the ecosystem and environmental quality, and economic benefits–both health and nonhealth related. Harms include all negative consequences from implementing the intervention. This includes human and environmental health risks and the economic costs that may result from implementing the intervention considered across the specific populations of interest and within marginalized populations.

##### Signaling Questions and Data Considerations

We developed five signaling questions for this criterion (Table S2, Supporting Information):

- What are the estimated human and environmental health benefits of the intervention and alternative options?
- Which health harms will likely be reduced with the intervention, and will they be reduced to the same extent across all populations?
- Are there human and environmental health harms/risks from the intervention and alternative options?
- What are the costs of the harm from the intervention and who bears them?
- What are the costs to implement the intervention and who bears them?

There are multiple approaches to quantifying health benefits, both their extent and the associated dollar valuation.^54^ Government agencies have developed guidelines around best practices, including different approaches to estimate valuation.^54^

There are two other important components of estimating health benefits. The first is the quantification of all potential human and environmental health benefits, including noncancer outcomes, that are frequently unquantified in current regulatory analyses of toxic chemicals and any health outcomes with evidence that is uncertain (e.g., “suggestive evidence”).^55^ The second is the cobenefits, which are reductions in harmful exposures other than those targeted by the intervention. These must be considered as part of any benefit-cost analysis and are critical in the development of health-protective policy. For example, there were substantial cobenefits to EPA’s 2012 Mercury and Air Toxics Standards (MATS) for power plants, as they significantly reduced fine particle emissions in addition to reductions in mercury emissions.^56−58^

When considering the costs of implementing an intervention, any estimates of economic costs calculated by the polluting industry must be rigorously and transparently scrutinized.^59,60^ Research shows that polluting industries consistently overestimate the costs of mitigation, while exaggerating the harms to the economy.^61−64^

Marginalized, impacted communities are often told that proposed mitigation or remediation are not possible due to cost or feasibility issues or because of impact on the economic stability of their community, including that they may benefit from jobs polluting facilities create.^59,61−64^ The proposition that removing or regulating these facilities could result in an increase in unemployment and poverty^65,66^ is a narrative often put forth by polluting companies and should be scrutinized. For example, analysis of the Clean Air Act shows it “has been a modest net creator of jobs through industry spending on technology to comply with it” and demonstrates such narratives are often false^64^ and thus must not be used as justification for failing to regulate polluting industries.

Another important consideration for this criterion is that accounting of benefits and harms may yield differential gains in net benefits–defined as the overall gains in health and economic savings resulting from an intervention compared to its costs–depending on the context. For example, an intervention might not show significant net benefits in the overall population; however, it may have net benefits for marginalized groups. Thus, the analysis of the distribution of benefits is important and informs this and the other criteria (e.g., Environmental Justice). It is critical to ensure that the intervention does not increase or contribute to inequitable impacts.

In the case study of PCE, the most sensitive end point from the EPA’s Final Risk Evaluation for PCE was neurotoxic effects including decrements in visual memory function, which can occur in certain neurodegenerative diseases like Parkinson’s disease and multiple sclerosis, and which may have solvent exposure as an etiological component.^35^ In order to determine the health benefits of the proposed intervention (sub criteria) in the most highly exposed and susceptible populations (i.e., fenceline communities and workers, while also taking account of additive and/or synergistic risks of the most impacted) the Decision-making panel could apply the WHO methodology^67^ and a recent analysis by Nielsen et al.^16^ to determine a workplace exposure value to protect workers and emissions limitation necessary to achieve annual average ambient concentrations to a level that eliminates unreasonable risk (e.g., 1–10,000 or 1-in 100,000 risk). The health benefits, including all noncancer outcomes and potential cobenefits from reducing exposure to PCE to this level are to then be quantified (data consideration and needs) (Figure 4, Step 2, ‘Maximizing Benefits & Harms’).

#### Sociocultural Acceptability, and Feasibility

##### Definition and Description

Sociocultural Acceptability and Feasibility evaluates factors within society that determine the most culturally sensitive, acceptable interventions, which thus may be most effective and sustainable in the long term.

##### Signaling Questions and Data Considerations

We developed five signaling questions for this criterion (Table S2, Supporting Information):

- How do affected communities/populations perceive the exposure and/or related health risks?
- What do affected communities/populations think of the proposed intervention, including its: effectiveness; unintended effects; effect across populations; feasibility of implementation
- Do impacted community members propose any changes to the intervention?
- Which cointerventions may be needed to overcome challenges associated with acceptability or feasibility?
- What are funding and infrastructure needs to overcome historical failures to provide the necessary resources to protect impacted communities?

Impacted communities, including rights and title holders and other stakeholders, may have synergistic priorities that impact what is valued, or priorities may be conflicting. There are also systematic power imbalances across these groups.^68^ It is important, therefore, to examine and document which communities are currently and historically most affected by exposures of concern and then prioritize those communities when assessing sociocultural acceptability of the potential intervention. Groups formulating decisions must also consider the impacted communities, including rights and title holders, and decision makers’ knowledge, beliefs, values, and interests, be these political, economic, symbolic, or otherwise defined.

It is essential to understand the acceptability of a proposed intervention to impacted communities and consider the response from those groups, which may affect the implementation of the intervention.^26^ As discussed throughout, this underscores the key importance of communities being collaboratively involved throughout decision and discussion processes; we believe that decisions should not *happen to* communities, but rather *happen with and in* communities.

The feasibility of implementing an intervention is also an important consideration for decision-makers, encompassing issues related to the existing infrastructure, resource needs and availability, accessibility, convenience, and potential disruptions to the lives of impacted persons.^68^ An assessment of feasibility may include the financial resources, the technological complexity of the intervention, and whether it is sustainable and entails potential legal, ethical, or bureaucratic barriers.^26,69^ Although a proposed intervention may be infeasible based on an assessment of past funding, currently available resources, or other institutional barriers, that does not mean a recommendation should not be made as those factors may be reflective of past environmental injustices that must be addressed.^68^

Feasibility assessment must include an examination of the potential role and power of industry and corporations to both control the narrative and resist change. One of the purposes of the framework is to enable decision-makers to disentangle and prioritize consideration of health, social, environmental, and racial justice from profit considerations. Therefore, industries’ imperatives to prioritize profits should be visible, understood, and directly addressed including the reliability of their commissioned data and research. This can be facilitated by narrating what is to be gained or lost by industry, drawing on their own narratives, their actions, and other reasonable understandings: what are they doing and why?

In the case study of PCE, fenceline communities and workers should be consulted on the effectiveness of the proposed interventions to reduce PCE exposure levels to protect their health, any unintended effects of the interventions, and how feasible it will be to implement for these groups. As these interventions will require technological controls to be put in place by the facilities processing PCE, these communities will not be directly impacted by feasibility considerations but will still be informed of what will be required for the intervention to be implemented. Support/funding by the facility for ongoing monitoring and stronger oversight and involvement by community organizations as part of the intervention must be considered.

## Discussion

The E2DFEH aims to provide a structured process for ensuring that all relevant factors in the decision-making process are transparently evaluated and deliberated on when developing recommendations for interventions to mitigate or prevent hazardous environmental exposures. The framework can be used for decision-making at the global, national, or local level. This framework could be used by community leaders, legislators, or regulators, among others, that convene scientific committees/decision-making panels. Our framework foundations and criteria are grounded on well-established principles articulated by the Montreal Protocol (Essentiality)^24^ WHO/UN (Human Rights),^37,38^ EPA (Environmental Justice and Maximizing Benefits Reducing Harms (WTP)),^46,55^ and E.O. 14096 on *Revitalizing Our Nation’s Commitment to Environmental Justice* (Environmental Justice and Sociocultural Acceptability, and Feasibility).^47^

### Implementation of the E2DFEH Framework

The E2DFEH should be used from the very beginning of the decision-making process–i.e. when framing the approach to select interventions, as it can require some time to identify, collect, and assess the data and evidence needed for each of the criteria. Typically, in environmental health, exposures are ongoing simultaneously with the decision-making process, which means that a longer time to identify an optimal recommendation can delay intervention to protect health. There may be negative consequences with taking an action that turns out to be ineffective or suboptimal. However, there can also be grave consequences with failing to take timely action,^70,71^ as exemplified in the European Environment Agency (EEA) report entitled “Late Lessons from Early Warnings.” Thus, health-protective actions should be taken by governments (at various levels responsible for protecting impacted communities) to mitigate or prevent exposure, while the data and evidence are collected and recommendations formulated. Using a rigorous process should not mean failing to act when a community is experiencing ongoing harm from hazardous environmental exposures.

The scientific committees/decision making panels tasked with developing intervention recommendations need to include individuals with a broad range of skills and experiences necessary to operationalize the framework, including environmental health, environmental justice, evidence synthesis, guideline methods, clinical medicine, law, and economics, and with experience in working at the jurisdiction (level of government) for which the recommendations are being developed and local community organizing, feedback, and advocacy.

Many communities that experience historic and persistent disparities in environmental exposures are often marginalized and unrepresented in public processes that directly impact their health and quality of life. Even when they can overcome the substantial barriers to participation, their voices are often silenced or diminished.^72^ Representatives of impacted communities therefore should play an important role on scientific committees/decision-making panels in selecting and prioritizing subcriteria; identifying relevant sources for data and evidence; and as an essential source of knowledge to inform criteria. Community representatives can represent specific geographic communities or communities defined by age, race, ethnicity, and income or other characteristics that may have experienced historical racism or disproportionate harm from hazardous environmental exposures.

To foster trust and engender systemic solutions, there must be transparency and meaningful engagement and collaboration with affected communities, including representation on the committees that propose interventions and formulate recommendations.^59,73^ Affected communities share crucial insights on the lived experience of systemic harms, the dynamics of how the system operates to harm them, and therefore how that might be disrupted and changed. Examples include informational interviews and ethnographic research about community members and their needs, values, and preferences with respect to potential interventions. By documenting their lived experiences and integrating qualitative research with quantitative data, we can center decision-making on communities that are most affected. Affected communities can thus provide guidance on potential solutions and on the acceptability and feasibility of implementing proposed interventions. Lastly, members of affected communities are critical for providing feedback on the success of interventions in reducing health inequity.

When developing these relationships, participatory and transformative approaches are required which recognize the health assets of individuals and communities affected by environmental contamination.^74,75^ Further, acknowledging the diverse types of knowledge communities may provide about toxicity and that all kinds of information are valid for informing interventions that are intended to protect them is critical.^76^ The field of Health Impacts Assessment (HIA) provides helpful guidance and case studies of effective interactions between policy makers and affected communities.^77^ To allow community members to contribute actively to research that informs solutions for the community, approaches that encourage humanistic ways of doing science are required and have great potential to address environmental justice issues.^78−81^

A recent U.S. National Academies of Sciences, Engineering, and Medicine (NASEM) report used such an approach when developing recommendations on how communities and individuals exposed to PFAS (Per- and polyfluoroalkyl substances) could be best served by clinicians though the use of PFAS blood testing.^68^ The NASEM prioritized the needs and preferences of these individuals in the formulation of the recommendations.^68^ We will use this approach for community engagement as a blueprint in the implementation of the E2DFEH.

### Potential Challenges in Using the Framework

Data on baseline exposure and risks for each highly exposed and vulnerable population may not be available or feasible to collect, depending on the scenario and resources available to decision-making panels; therefore, modeling or extrapolation from other populations/settings may need to be considered. Additionally, indirect evidence of the effectiveness of interventions may be needed when little or no evidence regarding interventions for a specific environmental exposure of concern is available. In such cases, mandatory monitoring (bio and ambient) and other continuous, subsidized feedback by the impacted communities on risks are required and all paid for by the facility/industry. Further, community representation in the decision-making process may not always be possible. In such instances, an explanation for the lack of representation must be provided, and relevant data must still be considered.

There is a need for government agencies such as the EPA to identify disparities in risks and health outcomes by social determinants of health (e.g., race and ethnicity and socioeconomic status). There are existing efforts within EPA to expand assessments of exposures and risks of hazardous agents, with subgroup comparisons to identify inequities.^82−87^ Without such federal action, decision-making panels may not have the necessary data for optimal decisions and actions.

Additional challenges may include identifying impacted communities who can meaningfully contribute to the decision making process; interpretation of the criteria, subcriteria, and signaling questions by different decision makers and guideline panels; and assessing the quality of the evidence, which may include a range of evidence for evaluation. We aim to address some of these challenges through the conduct of a pilot case study (see below “Next Steps”). Finally, determining the essentiality of a chemical’s use for health, safety, or the functioning of society will require data free from bias and informed by expert input, including from workers or communities impacted by the use of the chemical of concern throughout its life cycle, scientists, and health professionals. Manufacturers, distributors and sellers may provide necessary information and data on a chemicals function; however, strict conflict of interest policies are required to ensure any entities with a vested financial interest in the determinations are excluded from the decision-making process on essentiality to minimize bias.^23^ Confidential Business Information (CBI) must be limited given the community interest and risks, and industry must provide added, specific justification that protection of this information from disclosure will substantially affect its competitive position. Finally, although challenging the states of Maine and Minnesota have both successfully implemented this essentiality approach to eliminate use of PFAS “forever chemicals.”^88,89^ In 2021 Maine passed a law to phase out all uses of PFAS in products unless the state determined the use of PFAS is “currently unavoidable,” which it defined as when there is no safer alternative to PFAS in the product and the product itself is necessary for the health, safety or functioning of society.^88^

### Strengths and Limitations of the Approaches Used to Develop this Framework

We used a comprehensive, rigorous, inclusive, and iterative approach to develop E2DFEH, thus supporting the validity and utility of the new EtD framework.

We reviewed the existing EtD frameworks used by a range of organizations. The Steering Committee had broad and in-depth expertise in a wide range of relevant topics, and feedback from a diverse group was solicited.

There are limitations to our framework development process. Our review of existing EtD frameworks and organizations was targeted and not fully comprehensive. It is possible that we missed notable frameworks and decision criteria.^90^ While we made significant efforts to include all relevant perspectives and expertise throughout the process, we may have missed informative perspectives.

### Next Steps

The E2DFEH was developed through the lens of chemical policy and regulation in the U.S., and we will first apply and evaluate the framework in the context of chemicals regulated at the federal level in the U.S. We plan to conduct a pilot case study to develop recommendations to mitigate or prevent harms from a chemical exposure evaluated by the EPA under the TSCA.^91^ Through this process, we will amend and adapt the framework as necessary, following which we will apply it to a diverse spectrum of environmental issues globally and encourage other groups to use the framework and provide feedback.

UCSF PRHE used a similar, iterative approach to test and develop the Navigation Guide method for the hazards of chemical exposures.^28−32,92,93^ This process led to refinements and facilitated the dissemination and uptake of the method across the U.S.^94^ and internationally in partnership with the International Labor Organization (ILO) and WHO, to estimate the global burden of disease from various occupational risk factors.^95−97^ We envisage a similar approach with the E2DFEH: partnering with U.S. state agencies such as the California EPA, federal agencies such as the EPA, and international agencies such as WHO will provide opportunities for testing and application of the framework across levels of jurisdiction.

E2DFEH is a tool to help decision-makers in environmental health, including community leaders, legislators, and regulators, among others, to implement a transparent process for developing recommendations for interventions to mitigate or prevent exposure from environmental exposures of concern. The E2DFEH is intended to facilitate decisions that are equitable, transparent, and inclusive, centering and amplifying the voices of those who are the most impacted by hazardous exposures. All decisions are underpinned by the *Foundations* of Essentiality, Human Rights, and the Quality of the Evidence. The *Criteria* of Environmental Justice, Maximizing Benefits and Reducing Harms, and Sociocultural Acceptability and Feasibility are evaluated and supported by relevant evidence. Through every step of identification of exposures and issues of concern, data gathering, and decision-making, community stakeholders are active participants. Application of the framework in case studies in real-world settings, followed by refinement as indicated, will help to ensure that the E2DFEH meets the goal of a structured and transparent process that allows for decision making with a range of scientific information and considers important factors for making recommendations on interventions in a timely way to advance health and health equity.

## Supporting Information Available

Tables for the Navigation Guide Evidence-to-Decision Framework for Environmental Health Foundations and Considerations and Criteria, Sub-Criteria, Signaling Questions and Considerations. This work was supported by The JPB Foundation (G-2022–3608). JEJ, Supported in part by NIH 5P2CES03343. Max T Aung conducted this work while he was at the Program on Reproductive Health and the Environment, Department of Obstetrics, Gynecology, and Reproductive Sciences, University of California, San Francisco (PDF)
