# Supplementary material for: Development of the Navigation Guide Evidence-to-Decision Framework for Environmental Health: Version 1.0
Source: Environ Sci Technol. 2025 Feb 27;59(9):4230–44. doi: 10.1021/acs.est.4c08063 (PMC11912317; doi:10.1021/acs.est.4c08063)
Supplement: Supplementary file 1 — es4c08063_si_001.pdf [file es4c08063_si_001.pdf]

Development of the Navigation Guide Evidence-to-Decision Framework for Environmental Health: Version 1.0

Authors:
Nicholas Chartres, Max T. Aung, Susan L. Norris, Courtney Cooper, Lisa A. Bero, Roger Chou, Devon C. Payne-Sturges, Wendy E. Wagner, Jessica W. Reyes, Lisa M. Askie, Daniel A. Axelrad, Deysi Flores Vigo, Jill E. Johnston, Juleen Lam, Kegoeve E. Nachman, Eva Rehfuess, Rachel Rothschild, Patrice Sutton, Lauren Zeise, Tracey J. Woodruff

Supporting Information: Navigation Guide Evidence-to-Decision Framework for Environmental Health Foundations and Criteria, and hypothetical example of how the Framework would be operationalized when developing intervention recommendations

Summary: 10 pages, 0 figures, 2 tables

Table S1. Navigation Guide Evidence-to-Decision Framework for Environmental Health: Foundations and Considerations

Foundation	Considerations
Manufacturers, distributors, and sellers may provide essential information and data on a chemicals function; however, strict conflict of interest policies are required to ensure any entities with a vested interest in the determinations are excluded from the decision-making process on essentiality to minimize bias.<br>Data that are produced by those with a financial stake in the outcome, including the polluting industry, should be carefully evaluated, and considered at risk for bias.	
**Essentiality**<br><br>Identifying whether the exposure of concern (e.g., chemical agent) is considered essential for health and safety or for societies to function.	Three questions are considered to determine whether an exposure of concern (e.g., chemical agent) is essential for a particular use:1) Is the function of the chemical necessary for the product or use? 2) Is the use of the chemical the safest feasible option? 3) Is use of the chemical justified because such use in the product is necessary for health, safety, or for society to function?[1]<br><br>For efficiency, the question that can be most easily answered 'no' should be addressed first, eliminating the need to answer the other two questions and to conclude the process given that all three must be "yes" to continue to consider the chemical/product.<br><br>Only if all three questions are "yes" is it considered an essential use, otherwise it is not an essential use and should be substituted or discontinued.
**Human rights**<br>Human rights are rights inherent to all human beings, regardless of race, sex, nationality, ethnicity, language, religion, or any other status.[2] All environmental interventions and data synthesis must be guided by ethics, including respect for human rights and the moral rights that flow from them.	For environmental health interventions, the most important human right is the right to live in a clean, safe and healthy environment. The moral rights that this human right implies in most contemporary societies include the rights of equal/equitable access to clean air, water, sufficient health-promoting food, health, and the value of "lived experience."

**Quality of evidence**<br>The quality (or certainty) of evidence reflects the evaluation and summary of confidence in the evidence base. Quality of evidence is a continuum which should be articulated.	Optimal approaches to gathering and evaluating the evidence differ for each of the three framework criteria. The quality of the body of evidence relevant to each framework criterion should be evaluated with validated tools and approaches when available.<br><br>Decisions can be made using any level of evidence quality depending on the context of the decision.<br><br>There may be little or no direct evidence regarding interventions for a specific environmental exposure of concern, however, indirect evidence on the effectiveness of interventions for other exposures of concern may be informative.<br><br>Rehfuess et al., 2019 (Table 3) provides an overview of validated tools and approaches to evaluate the quality of the body of evidence relevant to each framework criterion that should be considered for use with the Navigation Guide Evidence-to-Decision Framework for Environmental Health.[3]

Table S2. Navigation Guide Evidence-to-Decision Framework for Environmental Health Criteria, Sub-Criteria, Signaling Questions and Considerations

All criteria are relevant for all environmental health interventions and policies. Sub-criteria should be discussed by the decision-makers as to which are most relevant and if how evidence should be collected to inform these. The signaling questions are conceptualized to help organize evidence gathering and synthesis for making recommendations.

Criteria	Sub-criteria	Signaling questions	Considerations
**Environmental justice**<br><br>Evaluates an intervention's capacity to overcome historic and persistent disparities in environmental exposures, the risks and health effects based on social factors such as race/ethnicity, education and income, among others and to help reduce current health inequities within and across affected populations.	• Impact of the environmental hazard (exposure, risk, and health effects) across different populations.<br>• Impact of the intervention on reducing health inequity across different populations<br>• Distribution of benefits and harms of an intervention across different populations.	• What is the historical and current distribution of environmental exposures and non-environmental stressors (e.g., socioeconomic status, racism/discrimination, immigration status) affecting different subgroups?<br>• What are the cumulative effects of all environmental exposures and non-environmental stressors?<br>• What is the expected/estimated reduction in exposure, risk, and health effects from the hazardous exposure following implementation of the intervention in subgroups that have been historically marginalized?<br>• How does the proposed policy address cumulative exposures and effects?	• Data are needed on the historical and baseline exposures and risks, and the distribution of benefits and harms of a intervention within and across affected populations.<br>• Factors that influence risk should be accounted for in any risk calculations, including non-environmental stressors: intrinsic factors (e.g., pre-existing disease, life stage, reproductive status, age, sex, genetic traits, health status) and extrinsic factors (e.g., geography, socioeconomic status, racism/discrimination, culture, immigration status, workplace), and other environmental exposures (e.g., chemicals).<br>• Quantitative estimates can be used to inform this criterion, however, there may be major data gaps for some

		• Will the expected distributional consequences of the intervention (including as part of any analysis of benefits and harms) appropriately benefit and not inappropriately burden disadvantaged, vulnerable, or marginalized communities?	communities and for some environmental hazards of concern. In these cases, consideration should be given to using indirect evidence from related populations and settings or to use qualitative data.<br>• Community-based, qualitative data can be leveraged to inform environmental justice issues and specific community data to inform broader context is appropriate.[4] Examples include informational interviews of, and ethnographic research about, community members and their needs, values, and preferences with respect to interventions.<br>• Strong justification would be needed for any intervention that maintains or increases inequities.
**Maximizing benefits and reducing harms**<br><br>Evaluates the immediate and long-term benefits and harms of the proposed intervention.	• Health benefits and harms of intervention.<br>• Positive and negative effects on ecosystems and	• What are the estimated human and environmental health benefits of the intervention and alternative options?<br>• Which health harms will likely be reduced with the intervention, and will they be reduced to the same extent across all subpopulations?	• All health benefits need to be considered including non-cancer outcomes and any health outcomes with evidence that is uncertain.<br>• One approach to benefits analysis used by the U.S EPA is willingness to pay (WTP). The goal of benefits analysis is to estimate the impacted

Benefits include reduction of disease, increased quality of life, positive effects on the ecosystem and environmental quality, and economic benefits - both health and non-health related. Harms include all negative consequences from implementing the intervention. This includes human and environmental health risks that may result from implementing the intervention and the economic costs of implementing the intervention, considered across the specific populations of interest and within marginalized subpopulations.	environmental quality.<br>• Financial savings on health and reduction of disease.	• Are there human and environmental health harms/risks from the intervention and alternative options?<br>• What are the costs of the harm from the intervention and who bears them?<br>• What are the costs to implement the intervention and who bears them?	individual's (or community's) total WTP.[5] WTP is defined as the greatest amount individuals (or in this instance, communities) are willing to pay in income to achieve these benefits, and not be any worse off. WTP for improved health benefits includes the quantification of avoided health care treatment costs, pain and suffering, other impacts of quality of life, and losses in productivity.<br>• Generally, WTP is higher for reducing risks of more severe health outcomes (e.g., chronic health conditions in children, cardiovascular disease, and cancer), therefore an outcome with uncertain evidence may provide greater benefits than an established "known" relationship on a less serious health outcome or an outcome with fewer expected cases avoided. Highly impacted communities will play an important role in identifying and prioritizing a set of outcomes – both benefits and harms.<br>• "Co-benefits", which are reductions in hazardous exposures other than those

			targeted by the intervention, must be quantified.<br>• Net benefits may vary with the context: e.g., total population versus marginalized subgroups.<br>• Considerations of who pays the costs of the implementation of an intervention should also be included in the analysis for this criterion: for example, the industries that may be the source of the harmful exposure of concern.
**Sociocultural acceptability and feasibility**<br><br>Evaluates factors within society that determine the most culturally sensitive, acceptable interventions, which thus may be most effective and sustainable in the long term.	• Sociocultural acceptability of the intervention to communities and populations most affected by the environmental hazard.<br>• Feasibility of implementing the intervention in communities and populations most affected by	• How do affected communities/populations perceive the exposure and/or related health risks?<br>• What do affected communities/populations think of the proposed intervention, including its: effectiveness; unintended effects; effect across sub-populations; feasibility of implementation<br>• Do impacted community members propose any changes to the intervention?	• Critical to consider affected communities' baseline knowledge of and concerns about the environmental hazard when evaluating this criterion.<br>• The greater the acceptability of an intervention by community members most burdened by the exposure of concern, the greater the likelihood of a general recommendation in favor of the intervention.<br>• When an intervention with net health benefits is considered less acceptable by community members in-depth discussions must be conducted with

	environmental hazard.	• Which co-interventions may be needed to overcome challenges associated with acceptability or feasibility?<br>• What are funding and infrastructure needs to overcome historical failures to provide the necessary resources to protect impacted communities?	impacted communities to understand concerns and explore modifications to the intervention to optimize the intervention.<br>• Although a proposed intervention may be infeasible based on an assessment of past funding, currently available resources, or other institutional barriers, that does not mean a recommendation should not be made as those factors may be reflective of past environmental injustices that must be addressed.<br>• Feasibility must consider environmental justice to guide policy makers to allocate necessary resources and investments to address historical disparities in exposures and health and be the catalyst for institutional changes.[6]<br>• Feasibility assessment must include an examination of the role and power of industry and corporations to both undermine communication related to the intervention and resist change, and explicitly recognize that health decisions are to be made in an ethical

			manner independent of a profit motive.

References

(1) Bălan, S. A.; Andrews, D. Q.; Blum, A.; Diamond, M. L.; Fernández, S. R.; Harriman, E.; Lindstrom, A. B.; Reade, A.; Richter, L.; Sutton, R.; Wang, Z.; Kwiatkowski, C. F. Optimizing Chemicals Management in the United States and Canada through the Essential-Use Approach. *Environ Sci Technol* **2023**, *57* (4), 1568–1575. https://doi.org/10.1021/acs.est.2c05932.

(2) United Nations. *Human Rights*. https://www.un.org/en/global-issues/human-rights (accessed 2024-11-19).

(3) Rehfuess, E. A.; Stratil, J. M.; Scheel, I. B.; Portela, A.; Norris, S. L.; Baltussen, R. The WHO-INTEGRATE Evidence to Decision Framework Version 1.0: Integrating WHO Norms and Values and a Complexity Perspective. *BMJ Global Health* **2019**, *4* (Suppl 1), e000844. https://doi.org/10.1136/bmjgh-2018-000844.

(4) Nweke, O. C.; Payne-Sturges, D.; Garcia, L.; Lee, C.; Zenick, H.; Grevatt, P.; William H Sanders, I. I. I.; Case, H.; Dankwa-Mullan, I. Symposium on Integrating the Science of Environmental Justice into Decision-Making at the Environmental Protection Agency: An Overview. *American Journal of Public Health* **2011**, *101* (Suppl 1), S19. https://doi.org/10.2105/AJPH.2011.300368.

(5) McGartland, A.; Revesz, R.; Axelrad, D. A.; Dockins, C.; Sutton, P.; Woodruff, T. Estimating the Health Benefits of Environmental Regulations. *Science (New York, N.Y.)* **2017**, *357* (6350), 457. https://doi.org/10.1126/science.aam8204.

(6) National Academies of Sciences, Engineering, and Medicine; Health and Medicine Division; Division on Earth and Life Studies; Board on Population Health and Public Health Practice; Board on Environmental Studies and Toxicology; Committee on the Guidance on PFAS Testing and Health Outcomes. *Guidance on PFAS Exposure, Testing, and Clinical Follow-Up*; The National Academies Collection: Reports funded by National Institutes of Health; National Academies Press (US): Washington (DC), 2022.
